# CARDIOVASCULAR HEALTH: Exploring a Potential Link between BPA and Heart Disease

**DOI:** 10.1289/ehp.118-a116a

**Published:** 2010-03

**Authors:** Naomi Lubick

**Affiliations:** **Naomi Lubick** is a freelance science writer based in Zürich, Switzerland, and Folsom, CA. She has written for *Environmental Science & Technology*, *Nature*, and *Earth*

Most people in the United States are exposed to the plastic monomer bisphenol A (BPA), whether in plastic linings in food cans, containers made of hard plastic, or other plastics and foods containing BPA. Exposure to this suspected endocrine disruptor is illustrated by urine samples collected for NHANES (the National Health and Nutrition Examination Survey), for which the Centers for Disease Control and Prevention surveys thousands of adults and children. A new analysis of NHANES data published 13 January 2010 in *PLoS ONE* adds more evidence for an association between heart disease and higher exposures to BPA, even at the relatively low levels seen in the general population.

David Melzer, an epidemiologist at the University of Exeter, United Kingdom, and his colleagues first looked at NHANES data collected from 2003 and 2004; their results, published in the 17 September 2008 issue of *JAMA*, were the first to show an association between higher levels of BPA metabolites in urine and adverse adult health outcomes, including heart disease. The current analysis considered NHANES data from 2005 and 2006. Although the BPA levels in urine samples from this new group of people were lower by almost a third, the association remained between coronary heart disease and higher urinary BPA.

While this study may help inform future research, such cross-sectional studies “should not be used to demonstrate that a particular chemical can cause a particular effect,” said Steven G. Hentges of the Polycarbonate/BPA Global Group of the American Chemistry Council in a 13 January 2010 press release. And indeed, Melzer and his colleagues emphasize that because this study is cross-sectional—a snapshot in time instead of a long-term observational investigation—they cannot say whether BPA contributes to heart disease or if heart disease changes the exposure to or metabolism of BPA in adults. “What would really help is if industry and regulators could support independent studies [to examine] whether high BPA levels are present before any disease started,” Melzer says.

“Chasing human [data] is the way forward,” says Richard Sharpe, an endocrinologist from The Queen’s Medical Research Institute in Edinburgh who did not participate in the research. NHANES provides a robust data set, he says, and the repeatability of the association with a second survey group is positive. However, that replication is somewhat incomplete because not all of the associations found with BPA in the first study were found in the second.

“The more logical interpretation of the results as they stand at the moment is that they are looking at two variables that are associated with something else,” Sharpe says. Diet, for instance, is a major contributor to heart disease in the United States and also a major source of BPA exposure. “We should remember that heart disease develops over a long period of time,” Sharpe says, “so if BPA is involved causally, a cross-sectional study such as this [*PLoS ONE* report] cannot show this.”

Scott Belcher, a scientist at the University of Cincinnati, says the association in humans is “not super surprising in light of our animal studies and because we already know estrogens are related to various cardiovascular end points.” Belcher’s team reported at the June 2009 Endocrine Society annual meeting that BPA exposure *in vitro* in muscle cells and in whole hearts from female rodents leads to arrhythmias, and they recently received American Recovery and Reinvestment Act funds from the NIEHS to conduct further research on BPA in mice. Meanwhile, pending the release of an updated toxicology review for BPA, the Food and Drug Administration announced in January 2010 it is “taking reasonable steps to reduce human exposure to BPA in the food supply.”

